# Transmission of Chromosomal MDR DNA Fragment Encoding Ciprofloxacin Resistance by a Conjugative Helper Plasmid in *Salmonella*

**DOI:** 10.3389/fmicb.2020.556227

**Published:** 2020-09-18

**Authors:** Chen Yang, Kaichao Chen, Edward Wai-Chi Chan, Wen Yao, Sheng Chen

**Affiliations:** ^1^College of Animal Science & Technology, Nanjing Agricultural University, Nanjing, China; ^2^Shenzhen Key Lab for Food Biological Safety Control, Food Safety and Technology Research Center, Hong Kong PolyU Shenzhen Research Institute, Shenzhen, China; ^3^Department of Infectious Diseases and Public Health, Jockey Club College of Veterinary Medicine and Life Sciences, City University of Hong Kong, Kowloon, Hong Kong; ^4^State Key Lab of Chemical Biology and Drug Discovery, Department of Applied Biology and Chemical Technology, The Hong Kong Polytechnic University, Kowloon, Hong Kong

**Keywords:** *Salmonella*, ciprofloxacin resistance, chromosomal fragment, PMQR genes, conjugative helper plasmid

## Abstract

Resistance to ciprofloxacin, a treatment choice for *Salmonella* infections, has increased dramatically in recent years in particular in serotype *Salmonella* Derby with most of strains carrying chromosome-encoded multiple plasmid-mediated quinolone resistance (PMQR) genes. In this work, we discovered a conjugative plasmid, pSa64-96kb, in a *Salmonella* Derby isolate, namely Sa64, which could extract and fuse to a multiple drug resistance (MDR) DNA fragment containing two PMQR genes, *aac(6’)-Ib-cr*, and *qnrS2* located on the chromosome of the *Salmonella* strain. This process led to the formation of a new 188 kb fusion plasmid, which could be then subsequently transmitted to recipient strain Escherichia *coli* J53. The chromosomal MDR DNA fragment was shown to be flanked by one copy of IS*26* element at each end and could be excised from the chromosome to form circular intermediate, which was then fused to pSa64-96kb and form a single plasmid through IS*26* mediated homologous recombination. The role of IS*26* on enhancing the efficacy of fusion and transmission of this chromosomal MDR DNA fragment was further proven in other *Salmonella* strains. These findings showed that dynamic interaction between specific chromosomal fragment and plasmids may significantly enhance resistance development and transferability of mobile resistance-encoding elements among bacterial pathogens.

## Introduction

Non-typhoidal *Salmonella* represents the primary bacterial causes of food-borne gastroenteritis throughout the world (Gomez et al., 1997). Antibiotics are unessential for treating salmonellosis, yet it can be lifesaving in cases of serious or systemic infections (Hohmann, 2001). Ciprofloxacin has been the agent of choice in treatment of *Salmonella* infection in adults. However, mounting evidences indicated the increasing prevalence of non-typhoidal *Salmonella* isolates exhibiting multidrug resistance phenotypes has significantly compromised the efficacy of present strategies applied to prevent and administer diseases related to food-borne infections (Molbak, 2005). Quinolone and fluoroquinolone resistance in *Salmonella* have been low and are commonly associated with a single or double mutations in the *gyrA* and/or *parC* gene (Hooper, 2001; Chen et al., 2004). Plasmid-mediated quinolone resistance (PMQR) genes have been described in recent years, which contribute to the extension of low-level quinolone resistance in Enterobacteriaceae. Three patterns of PMQR determinates have been described to date: (1) the Qnr types, which are mainly classified into seven different families and encoded pentapeptide duplicate proteins binding to DNA gyrase through imitating double stranded DNA, avoiding the binding of fluoroquinolones to gyrase; (2) the *aac(6’)-Ib-cr*, a modified aminoglycoside acetyltransferase that could hydrolyze fluoroquinolones; and (3) the efflux pumps QepA and OqxAB. However, detection of these PMQR genes in *Salmonella* remains rare before 2009, with the first detection of *qnrA* and *qnrS* genes being reported in Europe (Gunell et al., 2009).

In recent years, the percentage of resistance to ciprofloxacin has grown drastically in both environmental and clinical collections throughout the world, particularly in China and surrounding regions (Wong et al., 2014b). An interesting phenomenon is that the majority of ciprofloxacin-resistant *Salmonella* isolates displayed relatively low resistance levels (MIC < 8 μg/ml) to ciprofloxacin, with the proportion of strains exhibiting high resistance (MIC > 32 μg/ml) being very small and mostly belonging to the serotype *S*. Indiana. Strikingly, a significant proportion of these ciprofloxacin-resistant strains did not carry any mutations in the target genes. Instead, various types of PMQR genes were detectable in these strains. The *oqxAB* and *aac(6’)-Ib-cr* genes are usually carried by the same isolate, which might be related to the increased frequency of ciprofloxacin resistance in nosocomial *Salmonella* isolates recently (Wong et al., 2014a). Other PMQR genes such as *qnrS* was also increasingly reported in *Salmonella* (Lin et al., 2015). These PMQR determinates are commonly detected in the chromosomes and non-conjugative plasmids in *S.* Derby. Recently, conjugative ciprofloxacin resistance has been reported, including (1) conjugative plasmids encoding PMQR genes, and (2) helper plasmids mediating transmission of non-conjugative plasmids harboring multiple PMQR determinants, which might accelerate the further dissemination of resistance to ciprofloxacin in *Salmonella* (Chen et al., 2018, 2019a,b).

Here, we report the characterization of an IncI1 type of plasmid, which can excise the chromosomal DNA fragment containing multiple PMQR genes encoding ciprofloxacin resistance and transfer it to other bacteria through conjugation. Such recurrent genetic events could significantly increase the transferability of ciprofloxacin resistance genes among *Salmonella* and other Enterobacteriaceae species, potentially leading to a sharp increase in ciprofloxacin resistance in a range of bacterial pathogens in the near future.

## Materials and Methods

### Strain and Phenotypic Characterization

*Salmonella* strains Sa64 and Sa79 were isolated from a pork sample in supermarket in Shenzhen, China, in 2014 as described in our previous study on *Salmonella* surveillance in Shenzhen, China (Chen et al., 2018). The strains were confirmed to be *Salmonella* by MALDI-TOF MS and serotyped with commercial antiserum (Difco, Detroit, MI, United States) through the Kauffmann–White scheme. Antimicrobial-resistance phenotypes of these strains against a variety of antimicrobial agents (Table 1) were conducted with agar dilution and explained in agreement with the CLSI guidelines (CLSI, 2016). Quality control strains were Staphylococcus *aureus* ATCC 29213 and *Escherichia coli* ATCC 25922.

**TABLE 1:** Genetic and phenotypic characteristic of ciprofloxacin-resistant *Salmonella* isolates Sa64, Sa79, and its corresponding transconjugants.

Strain ID	Species	ESBL/PMQR genes	Plasmids (~kb)	Mutation	MIC(μ g/ml)													
					AZI	AMK	CTX	CIP	KAN	OLA	STR	CRO	TET	CHL	NAL	MRP	AMP	STX
Sa64	*S.* Derby	*qnrS2-aac(6’)Ib-cr*	96	–	2	16	0.25	8	>128	64	64	0.12	>32	64	32	0.12	>64	>32
Sa79	*S.* Derby	*qnrS2-aac(6’)Ib-cr-oqxAB*	–	–	1	16	0.12	8	>128	64	32	0.06	>32	64	>64	0.12	>64	>32
Sa64-TC	*E. coli J53*	*qnrS2-aac(6’)Ib-cr*	188	–	1	4	0.06	8	>128	32	16	0.06	16	32	16	0.06	>64	16
J53	*E. coli*	*–*	–	–	1	≤0.5	≤0.015	0.015	≤0.5	4	2	≤0.015	0.5	1	2	0.03	2	4

*ND, not determined; ESBL, extended spectrum β-lactamase; PMQR, plasmid mediated quinolone resistance AMK, amikacin; AZI, azithromycin*;* CTX, cefotaxime; CIP, ciprofloxacin; KAN, kanamycin; OLA, olaquindox; STR, streptomycin; CRO, ceftriaxone; TET, tetracycline; CHL, chloramphenicol; NAL, nalidixic acid; AMP, ampicillin; MRP, meropenem; and SXT, trimethoprim/sulfamethoxazole.*

### Conjugation Experiments

Conjugation experiments were performed following a previous study (Li et al., 2015a) to test the transferability of the *aac(6’)-Ib-cr- qnrS2* bearing plasmid, using a sodium-azide-resistance *E. coli* J53 as the recipient. Briefly, overnight incubation of Sa64 and strain *E. coli* J53 were blended in a rate of 1:4 in Luria Bertani (LB) nutrient broth, which was then exposed to overnight culture on an LB agar media. The admixture was then disseminated on a selective Eosin Methylene Blue agar plate supplemented with ciprofloxacin (0.5 mg/L) and sodium azide (100 mg/L) to pick out transconjugants that had obtained the PMQR-carrying plasmid. And other conjugation experiments were performed in the similar procedure.

### S1-PFGE and Southern Hybridization

*Salmonella* Derby strains Sa64 and its corresponding transconjugants were subjected to S1-PFGE to obtain the length of the plasmids. Briefly, S1-nuclease was used to digest agarose embedded DNA at 37°C for 15 min. The restriction bands were dispersed by using a Chef Mapper electrophoresis system (Bio-Rad, Hercules, CA, United States) with 2.16 to 63.8 S pulse times in 0.5 Tris–borate-EDTA buffer at 14°C. DNA fingerprinting of H9812 was used as DNA dimension marker. The gels were subjected to GelRed staining, and DNA bands were imaged with UV transillumination (Bio-Rad). Southern bolt hybridization was conducted according to the manufacturer’s directions of the Detection Starter Kit II (Roche Diagnostics), using the digoxigenin-labeled *qnrS* gene probe.

### Plasmid Sequencing and Analysis

Whole genome sequencing was conducted to acquire comprehensive understanding of the ciprofloxacin PMQR genes harbored by Sa64, Sa79, and Sa64-TC with the Illumina HiSeq 2500 sequencing, Nanopore MinION long-read sequencing. Genome sequence was assembled with SPAdes 3.12.1 (Bankevich et al., 2012). Long contigs assembled from Nanopore was used to align and join contigs obtained from Illumina assembly with the CLC Genomics Workbench v10 (CLC bio, Denmark). The complete genome sequence was annotated using the RAST server (Overbeek et al., 2013). Multilocus sequence typing (MLST) service of the Center for Genomic Epidemiology was used to test Sequence type (ST) typing and screen PMQR-genes as described previously (Chen et al., 2007). EasyFig and BRIG were used to compare genome contents, and plot linear and circular plasmid maps, respectively. A polymerase chain reaction (PCR) assays was performed as described previously (Li et al., 2015b) to check the circular intermediate status of chromosomally encoded multiple drug resistance (MDR) fragment using primer sets listed in Supplementary Table 1.

## Results

**FIGURE 1:**
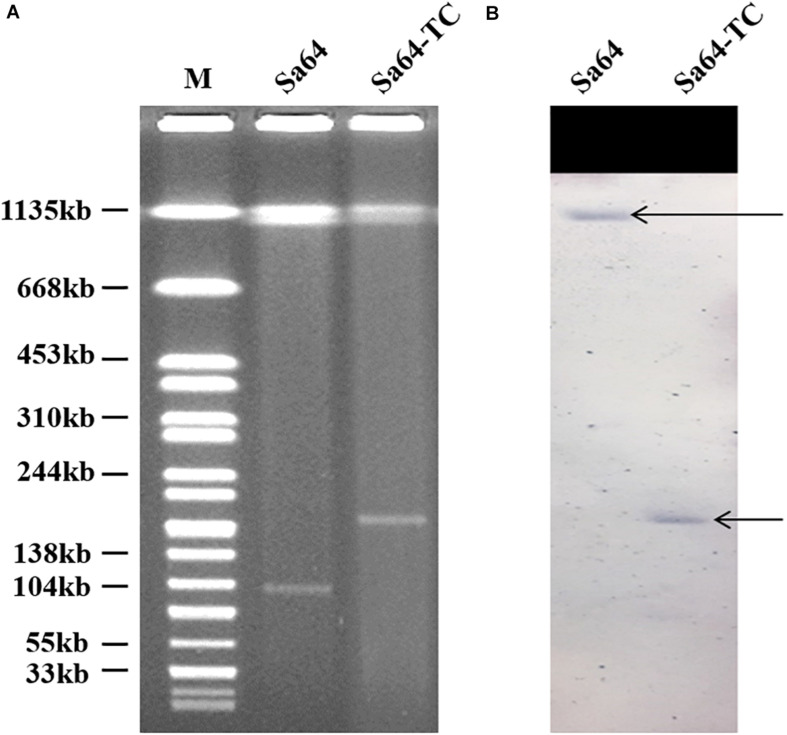
S1-PFGE of ciprofloxacin-resistant *Salmonella* strain Sa64, and its corresponding transconjugant*s.* **(A)** S1-PFGE patterns of *Salmonella* isolate Sa64 and its corresponding transconjugant; **(B)** Southern blot hybridization results of *Salmonella* isolate Sa64 and its relevant transconjugant. The arrow depicts the position of chromosomal DNA and plasmid band in which positive hybridization semaphore of the *qnrS* gene was detectable.

Our surveillance project on ciprofloxacin resistance in *Salmonella* in food samples collected from the wet markets and supermarkets in Shenzhen, China, allowed us to identify a *Salmonella* isolate that could transfer its phenotype of ciprofloxacin resistance *via* conjugation. S1-PFGE analysis was then conducted on this strain and its transconjugant showing that there was only one plasmid with the size of 96 kb in strain Sa64, whereas a larger plasmid of 188 kb was detectable in the transconjugant Sa64-TC, suggesting that the plasmid in strain Sa64 acquired an extra DNA fragment during the conjugation process. This finding prompted us to investigate the molecular mechanism underlying the conjugative transmission of ciprofloxacin resistance in *Salmonella*. Strain Sa64 was found to belong to ST40 and exhibited resistance to a wide range of antimicrobial agents, containing ciprofloxacin, kanamycin, tetracycline, and Sulfamethoxazole-methoxazole, but remained sensitive to cephalosporin and meropenem. The ciprofloxacin-resistance (Cip^*R*^) phenotype of this strain was able to be transferred through conjugation. Mutations were not detected in the four target genes, *parC*, *gyrA, gyrB*, and *parE*. PCR screening of known PMRQ genes showed that this strain harbored two PMQR determinants, *aac(6’)Ib-cr*, and *qnrS2*, which were also detectable in the transconjugants (Table 1). Southern hybridization using the *qnrS2* probe suggested that the *qnrS2* gene was located in the chromosome of strain Sa64 and the 188 kb plasmid in the transconjugant Sa64-TC, respectively, indicating that the ciprofloxacin-resistant genes were originally existed on the chromosome of strain Sa64 and then captured by the 96 kb plasmid and transmitted to *E. coli* J53 through conjugation (Figure 1). Interestingly, another Cip^*R*^ isogenic *Salmonella* strain, namely *S.* Derby Sa79, sharing the similar structure in both chromosome (Supplementary Figure 1) and chromosomal MDR DNA fragment (Supplementary Figure 2A) but not plasmid, does not show the potential ability to transfer its ciprofloxacin resistance to the recipient strain *E. coli J53* in this study.

**FIGURE 2:**
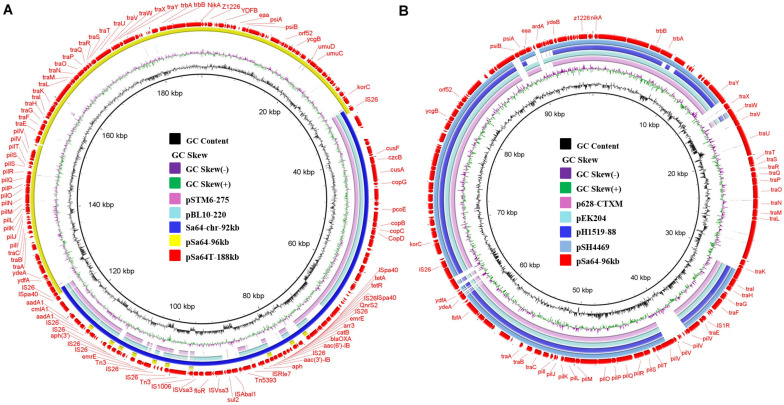
Circular alignment of plasmid pSa64-188kb and the pSa64-96kb. **(A)** The red circle depicts plasmid pSa64-188kb, which is used as reference; basic genetic loci in this plasmid are tagged. The light pink and green circle, respectively, indicate pSTM6-275 (275,801 bp, CP019647.1) and pBL10-220k (220,231 bp, CP025340.1), respectively; the circle in deep blue color represents DNA fragament Sa64-chr-92k from chromosomal of Sa64 and yellow circle represents pSa64-96kb in this study; **(B)** The light pink and blue circle represent plasmid p628-CTXM (85,338 bp: KP987217.1) and pEK204 (93,732 bp: EU935740.1), respectively; The blue circle with deep and light color, respectively, depicts pH1519-88 (88,678 bp: KJ484630.1) and pSH4469 (91,109 bp: KJ406378.1) in the NCBI database. The outmost circle in red color represents pSa64-96kb. Sequence was produced *via* combination of Illumina and PacBio sequencing data.

To investigate the mechanism underlying the transfer of ciprofloxacin resistance from Sa64 to *E. coli* strain J53, the complete sequences of the chromosome and the 96 kb plasmid in strain Sa64, as well as the ∼188 kb plasmid from the transconjugant Sa64-TC were obtained by using both Nanopore and Illumina sequencing platforms. The chromosome of Sa64 was found to be 4,824,198 bp in length with a Guanine and Cytosine (GC) content of 52.1%, and comprise 5,253 predicted coding sequences. BLANTN analysis showed that it exhibited high homology (92% coverage and 99% identity) to the chromosome of *Salmonella* strain ST350 (CP019407, recovered from a *Salmonella enterica* subsp. enterica serovar Borreze strain) isolated several decades ago from pig feces. Comparative genomic analysis showed that the chromosome of Sa64 had acquired an extra DNA fragment when compared to strain ST350 (Supplementary Figure 1). This extra DNA fragment was shown to be 92,690 bp in size and exhibit a GC content of 51.3%. BLAST of this DNA fragment showed that it displayed high homology (99% identity with 75% and 81% coverage, respectively) to other MDR-encoding plasmids such as pSTM6-275 (CP019647) and pbl10-220 (CP025340; Figure 2A), both of which were isolated from *Salmonella* strains in pig feces. These data suggest that this ∼92 kb DNA fragment in the chromosome of Sa64 might be acquired through integration of an MDR plasmid into the chromosome. Analysis of Nanopore reads of strain Sa64 has identified ten reads covering two fusion regions between chromosome and the ∼92 kb DNA fragment, which confirmed the presence of MDR plasmid in chromosome in strain Sa64 (Supplementary Figure 3). Sequence analysis showed that this DNA fragment contained a variety of antimicrobial resistance genes surrounded by different IS elements, such as *bla*_*OXA*_, *qnrS2, floR, aac(6’)-Ib-cr, tet(A)*, and *aph* genes, and some other resistance gene cassettes surrounded by diverse mobile elements. In this fragment, resistance genes that might contribute to the ciprofloxacin resistance phenotype were found to be located in a complex mobile element comprising various genes, with a structure of IS*26*-*aac(6’)Ib-cr-carB*-*arr3-emrE*-IS*6*-*qnrS2*-IS*26*, suggesting that IS*26* played a critical role in the formation of this mobile element and subsequent integration into the chromosome of strain Sa64, with the potential of being reacquired by other plasmids harbored by the host strain.

The plasmid harbored by *Salmonella* strain Sa64, designated as pSa64-96kb, was 96,571 bp in length, with GC content of 49.7%. BLASTN analysis indicated that this plasmid was 99% homologous to plasmid pS68 (Accession number: KU130396) of which was acquired from *E. coli* at 99% coverage. Plasmid pSa64-96kb carried IncI1 replicon together with the transfer proteins (*tra* locus), the pilus formation protein (*pil* locus), the shufflon-specific DNA recombinase (*rci*), and the *nikAB-trbABC* region encoding genes. It also showed homology (98.9% similarity, 72–76% coverage) to some other plasmids, including *E. coli* plasmid pEK204 (EU935740.1) and *Klebsiella pneumoniae* plasmids p628-CTXM (KP987217.1) and pH1519-88 (KJ484630.1), and a *Shigella sonnei* plasmid, pSH4469 (KJ406378.1), all of which contained a *bla*_*CTX–M*_ gene belonging to group 1 variant and contributed to worldwide transmission of ceftriaxone resistance in *Salmonella* (Figure 2B). The insertion sites of the *bla*_*CTX–M*_ variants were very similar on these plasmids (Supplementary Figure 2B). However, no antimicrobial resistance gene was carried by pSa64-96kb, and thus it could be considered as the prototype of such IncI1 plasmids. Additional *tra* genes responsible for conjugation were carried by pSa64-96kb but were replaced by diverse antimicrobial resistance genes in other IncI plasmids during plasmid evolution (Figure 2B and Supplementary Figure 2).

**FIGURE 3:**
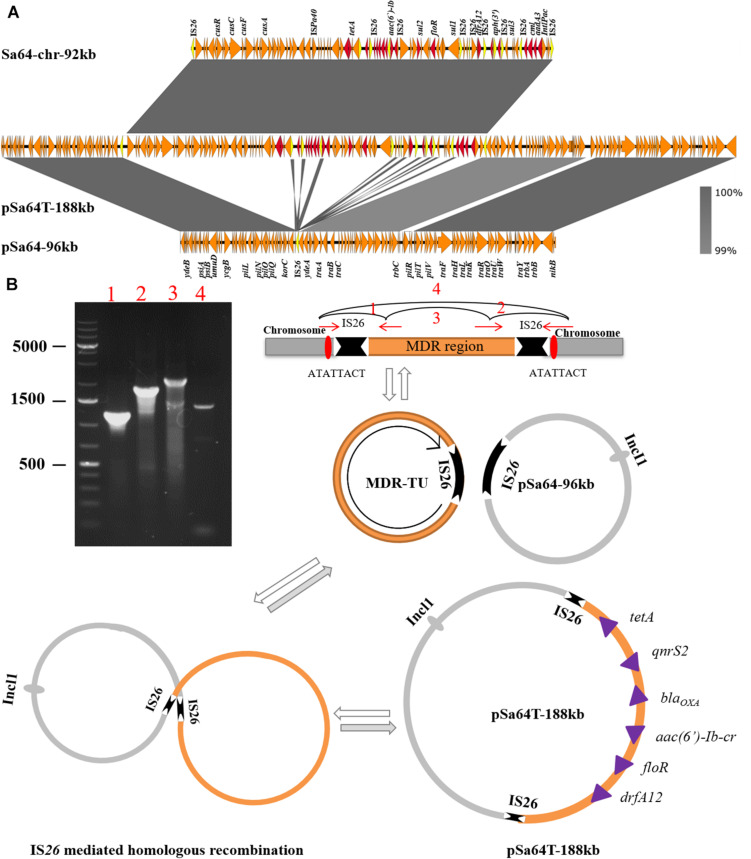
Mechanisms of plasmid recombination. **(A)** Structure alignment of two plasmids and the MDR region in the chromosome. Duplicated IS*26* elements are highlighted in yellow color and drug-resistance genes are depicted in red arrows; **(B)** Proposed IS element-mediated plasmid and drug-resistant chromosomal fragament fusion in Sa64, PCR products 3 and 4 confirm that TU could be dynamic existed in a circular intermediate form and also could be assembled in chromosome *via* replicative transposition event by the hot spot (ATATTACT). While the plasmid pSa64T-188kb is the cointegrate generated by homologous recombination bewteen TU and plasmid pSa64-96kb.

The plasmid recovered from the transconjugant S64-TC was a circular IncI1 plasmid of 188,966 bp, with 229 predicated coding sequences and, a GC content of 50.5%. It was designated as pSa64T-188kb. Sequence analysis showed that a ∼92 kb DNA fragment, (Sa64-chr-92kb) and the plasmid pSa64-96kb, which existed in the parental strain as separate entity, have merged and formed the fusion plasmid pSa64T-188kb though IS*26* (Figures 2B, 3A), which was subsequently transferred to the recipient strain J53 through conjugation. Taken together, our data could help explain the conjugative transmission of ciprofloxacin resistance in *Salmonella* Sa64 strain. The carriage of multiple PMQR genes [*aac(6’)Ib-cr* and *qnrS2*] in the MDR fragment located in the chromosome of Sa64 could confer resistance to ciprofloxacin (CIP MIC = 8 μg/ml), which was further confirmed by the increase of CIP MIC from 0.01 to 8 μg/ml after acquisition of conjugative plasmid pSa64T-188kb bearing this MDR fragment carrying *aac(6’)Ib-cr* and *qnrS2* in *E. coli* J53.

The molecular mechanisms underlying this DNA transmission and plasmid fusion were further investigated. Detailed analysis of the aligned sequences of these two plasmids and the chromosomal DNA fragments revealed that two copies of IS*26* were found to be flanked at both ends of chromosomal MDR fragment, Sa64-chr-92kb, and one copy of IS*26* on plasmid pSa64-96kb. These data suggested that the insertion of Sa64-chr-92kb to plasmid pSa64-96kb could be due to homologous recombination mediated by IS*26*. We then ask how the chromosomal DNA fragment could be integrated into plasmid by recombination. It has been reported that DNA fragment flanked by IS*26* could form circular intermediate known as a translocatable unit (TU; Harmer and Hall, 2015, 2016). To test if MDR Sa64-chr-92kb fragment could also form TU, four PCR assays using primers listed in Supplementary Table 1 were performed. PCR products 1 and 2 were successfully amplified with corrected sizes and sequences suggesting that these four primers were valid (Figure 3B). The amplification of product 3 as shown in Figure 3B was also obtained with right size and sequence, which aligned to the IS*26* sequence confirming that a TU has been formed in *S.* Derby Sa64 strain. In addition, PCR product 4 with size and sequence covering one copy of IS*26* and adjacent sequences suggested that some population of Sa64 carried one copy of IS*26* with the whole MDR fragment being excised out (Figure 3B). This data also indicated that the dynamic presence of TU and chromosomal encoded MDR fragment in a total population of Sa64. Analysis of product 4 sequence and sequences flanking MDR fragment on the chromosome allowed us to identify a hot spot (ATATTACT) located right outside of IS*26*, which implied that Sa64 obtained this MDR fragment through being attacked by IS*26* flanking the MDR region from other sources most likely from other plasmid at this hot spot on the chromosome. After confirmation of the presence of TU in Sa64, it is further speculated that this TU could undergo cointegration with pSa64-96kb to form pSa64T-188kb through homologous recombination at IS*26* region (Figure 3B).

## Discussion

Pathogenic bacteria such as *Salmonella* often contain target gene mutations and exhibit resistance to ciprofloxacin. Double and single mutations in *gyrA* and *parC* genes associated with ciprofloxacin resistance phenotypes in *salmonella* were shown to be consistent with previously reports (Chen et al., 2007). *Salmonella* isolates were rarely resistant to ciprofloxacin in the past decades mainly due to the low occurrence of *gyrA* double mutations. In 2005, quinolone resistance conferred by the acquisition of PMQR genes was first observed in *Salmonella*, while mutations in PMQR determinants only mediated quinolone resistance, but not resistance to fluoroquinolones such as ciprofloxacin (Ferrari et al., 2013; Wong et al., 2014a; Kim et al., 2016). The prevalence of ciprofloxacin resistance in *Salmonella* climbed sharply in several countries, especially China, with up to ∼ 30–40% resistance rate detectable among strains of specific serotypes (Lin et al., 2015; Pribul et al., 2017). These emerging ciprofloxacin-resistant *Salmonella* strains were found to harbor only a single *gyrA* mutation and an extrachromosomal PMQR gene, or several PMQR genes with no mutations. Apparently, the ciprofloxacin-resistance phenotypes of these isolates were attributable to the combined effects of the PMQR gene product, and/or target mutations because the PMQR gene products can drastically reduce the antimicrobial effect of fluoroquinolones *via* antibiotic efflux, competitive inhibition by binding of antibiotics, and inactivation with enzymes, resulting in emergence of high-level quinolone resistance strains without target gene mutations. However, ciprofloxacin resistance strains of *Salmonella* whose resistance phenotypes are transferable have rarely been reported, since the majority of PMQR genes were normally located in non-conjugative plasmid or the chromosome of *Salmonella* (Lin et al., 2015). However, the ciprofloxacin-resistance phenotype encoded by two types of conjugative plasmids has been reported in *Salmonella* previously (Chen et al., 2018), suggesting that ciprofloxacin resistance is now readily transferrable.

This study identified a chromosomal DNA fragment carrying PMQR and other drug-resistance genes that was assembled *via* plasmid-mediated integration into the *Salmonella* chromosome. Importantly, such chromosomal fragment may readily become transferable upon being captured by a conjugative plasmid in *Salmonella*. Emergence of such chromosome-derived, ciprofloxacin-encoding conjugative plasmids comprises a severe public health threat since ciprofloxacin is first option for curing life-threatening Salmonellosis. Comprehensive analysis of the genetic content among strains Sa64, Sa79, and ST350 showed that one insertion sequence, IS*26*, commonly co-occurred with antimicrobial resistance genes and were highly transferable between plasmids and the chromosome. The plasmid pSa64-96kb, which contains IncI1 replicon, was found to be an important resistance-encoding vector commonly harbored by various bacterial species. One part of this plasmid, the IncI1 replicon, may readily be fused with other drug-resistance genes at high frequency. Such fusion plasmids, such as pSa64T-188kb, exhibit a broader host spectrum and might help further expand the resistance profile of the host strain. It has been shown that plasmids are functionally dynamic, readily forming hybrid plasmids through recombination. Although the structure of Sa64-chr-92kb in the chromosome of the test strain was found to be stable *via* Nanopore long-reads sequencing. No TU of MDR fragment, Sa64-chr-92kb, could be detected with sequencing depth of 40 multiplicative, while it was shown that this type of TU was presented in some population of Sa64 by PCR amplification. This is probably due to the low number of this TU in total population of Sa64, which could not be picked up by Nanopore sequencing with low sequencing depth, which is consistent with the weak band of product 4 obtained by PCR. Plasmid cointegration is not rare among bacteria, and it has been reported to be related to spreading of antimicrobial resistance genes, such as cephalosporin resistance encoded by beta-lactamases genes (Leelaporn et al., 1996; He et al., 2015; Wong et al., 2016). For example, Harmer and Hall (2015, 2016) found that the insertion sequence IS*26* played a role in reorganizing plasmids in clinically isolated MDR bacteria *via* replicative transposition (Kim et al., 2016). Our recent work also reported that dissemination of IncI1 plasmids can integrate with various *bla*_*CTX–M*_ genes such as the *bla*_*CTX–M*_ group 1 and group 2 elements, contributing to an increasing prevalence of ceftriaxone resistance (Wong et al., 2016). These plasmids ranged from 75 kb to 100 kb in size and contained *bla*_*CTX–M*_ typical structures, IS*Ecp1-bla*_*CTX–M–*__14_-IS*903-iroN* or flanked by IS*Ecp1* element and a truncated *orf477* in up- and downstream regions, respectively. Our study further extends the role of this type IncI1plasmid as helper plasmid to fuse with chromosomal MDR fragment or MDR plasmid to help conjugate these non-conjugative elements to other bacteria with even broader host spectrum.

In conclusion, this study showed that dynamic interaction between specific chromosomal fragment and plasmids may significantly enhance resistance development and transferability of mobile resistance-encoding elements in bacterial pathogens. Mobile elements including both IS*26* and the IncI1 plasmid played important roles during the transmission process. IS*26* mediated homologous recombination of antimicrobial resistance genes with the IncI1 conjugative helper plasmid, during which a recombined fusion plasmid was generated. The fusion plasmid was transferred to a new host with helper of conjugative elements encoded by the IncI plasmid. Fusion between IncI1 type plasmids and various antibiotic-resistance gene-bearing mobile elements and chromosomal fragments is expected to bring about a sharp increase in the number of mobile resistance elements circulating among populations of bacterial pathogens, thereby posing a severe threat to the effectiveness of traditional antibiotics in treatment of infections caused by *Salmonella* pathogens. Meanwhile, such fusion events explain that bacteria may acquire antibiotic resistance genes from a route other than conjugation. Furthermore, such events will continue to produce novel plasmids conferring enhanced resistance phenotypes with expanded bacterial hosts.

## Data Availability Statement

The sequencing data of the plasmids and chromosome have been deposited in GenBank under the accession numbers pSa64T-188kb (CP034251), pSa64-96kb (CP034252), Sa64-chr (CP034250), and Sa79-chr (SGWG00000000).

## Author Contributions

CY collected the strain and finished the wet experiments. KC performed the sequencing and bioinformatics analysis. EC, KC, and SC participated in research design and manuscript writing. SC and WY supervised the whole project. All authors contributed to the article and approved the submitted version.

## Conflict of Interest

The authors declare that the research was conducted in the absence of any commercial or financial relationships that could be construed as a potential conflict of interest.
