# Supplementary material for: Transmission of Chromosomal MDR DNA Fragment Encoding Ciprofloxacin Resistance by a Conjugative Helper Plasmid in Salmonella
Source: Front Microbiol. 2020 Sep 18;11:556227. doi: 10.3389/fmicb.2020.556227 (PMC7530939; doi:10.3389/fmicb.2020.556227)
Supplement: Supplementary file 1 [file Data_Sheet_1.docx]

Supplementary Materials

**Transmission of chromsomal MDR DNA fragment encoding ciprofloxacin resistance by a conjugative helper plasmid in *Salmonella***

Chen Yang^1#^, Kaichao Chen^2#^, Edward Wai-Chi Chan^3^, Wen Yao^1*^, Sheng Chen^2*^

^1^College of animal science & technology, Nanjing Agricultural University, Nanjing, China;

^2^Department of Infectious Diseases and Public Health, Jockey Club College of Veterinary Medicine and Life Sciences, City University of Hong Kong, Kowloon, Hong Kong;

^3^State Key Lab of Chemical Biology and Drug Discovery, Department of Applied Biology and Chemical Technology, The Hong Kong Polytechnic University, Hung Hom, Kowloon, Hong Kong.

# contribute equally to the works.

*Corresponding authors: Sheng Chen, Tel: 852-34425782, Email: [shechen@cityu.edu.hk](mailto:sheng.chen@polyu.edu.hk); Wen Yao, [yaowen67jp@njau.edu.cn](mailto:yaowen67jp@njau.edu.cn)

**Keyword:** *Salmonella*, ciprofloxacin resistance, chromosomal fragment, PMQR genes, conjugative helper plasmid

**Supplementary Table S1. Primers used in this study.**

| **PCR Products** | **Primer** | **Size** | **Sequence (5′–3′)** |
| --- | --- | --- | --- |
| Product 1 | Sa64-1R | 1214bp | CTGGCGAAGACTCTCCGATG |
|  | Sa64-1F |  | AAGACAGAGCGGAACTCACC |
| Product 2 | Sa64-2R | 1898bp | AGACGAATATCCTGGCGCTG |
|  | Sa64-2F |  | CGTCAGTCCATTGGCTTTGC |
| Product 3 | Sa64-1R | 1375bp | CTGGCGAAGACTCTCCGATG |
|  | Sa64-2F |  | CGTCAGTCCATTGGCTTTGC |
| Product 4 | Sa64-1F | 2100bp | AAGACAGAGCGGAACTCACC |
|  | Sa64-2R |  | AGACGAATATCCTGGCGCTG |

**
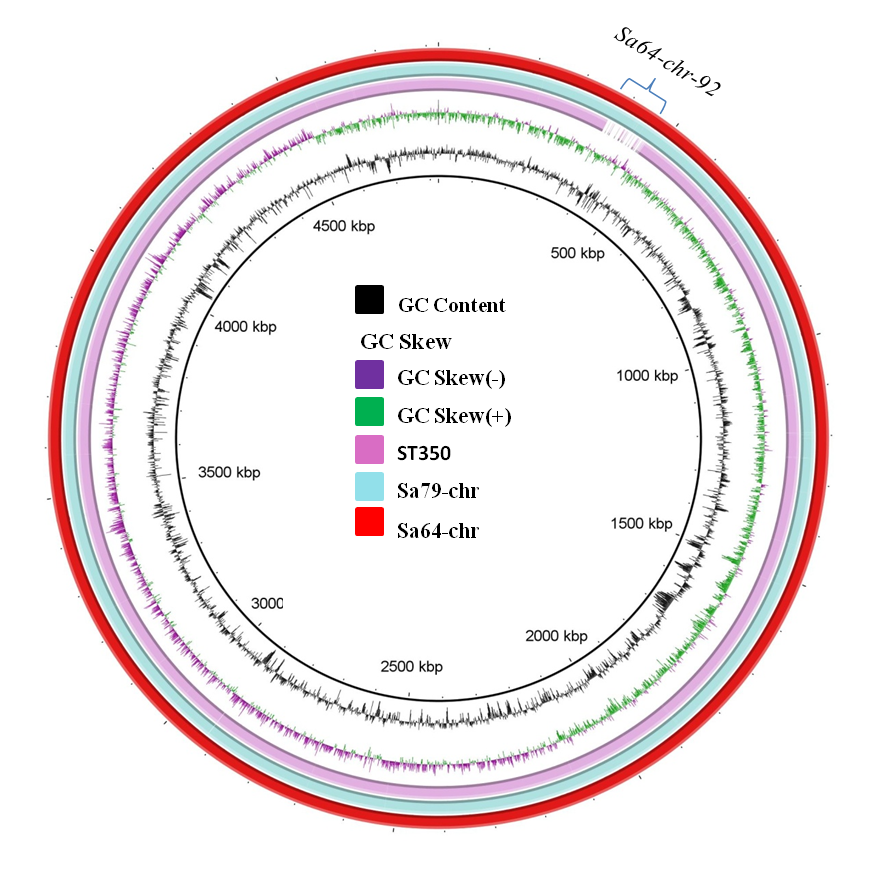
**

**Supplementary Figure S1.**  **Circular alignment of chromosome Sa64-chr, Sa79-chr and ST350 recovered from *Salmonella.* Derby** Comparative genomic analysis showed that the chromosome of Sa64 and Sa79 had acquired an extra DNA fragment when compared to strain ST350**.**


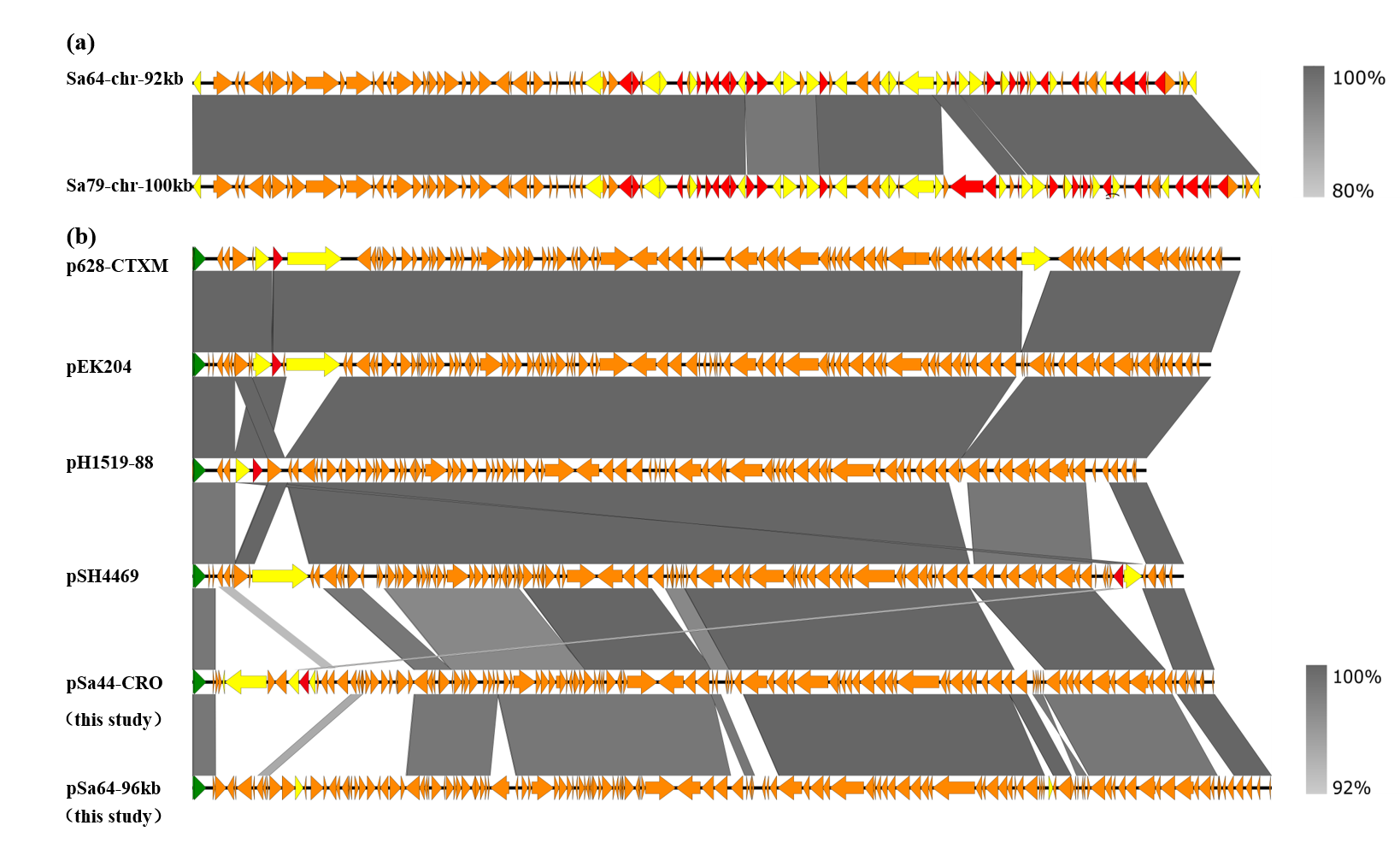


**Supplementary Figure S2.** (a) Structure alignment of mutiple drug resistance-encoding regions in the chromosome of Sa64 and Sa79, IS elements and drug resistance genes are highlighted in yellow and red color respectively, transport unit IS*26*-*OqxAB*-IS*26* located in the chromosome of Sa79, which is structurally different from Sa64. (b) Complete map of pSa64-96kb and its genetic similarity to other incl1 plasmids. Comparison of the pSa64-96kb sequence with the sequences of five other incl1 plasmid harbored different *bla*CTX-M  genes belonging to group 1 variant. Plasmids p628-CTXM (85,338 bp: [KP987217.1](https://www.ncbi.nlm.nih.gov/nuccore/KP987217.1)), pEK204 (93,732bp: [EU935740.1](https://www.ncbi.nlm.nih.gov/nuccore/EU935740.1)), pH1519-88 (88,678 bp: [KJ484630.1](https://www.ncbi.nlm.nih.gov/nuccore/KJ484630.1)),pSH4469 (91,109bp: [KJ406378.1](https://www.ncbi.nlm.nih.gov/nuccore/KJ406378.1)) ) and pSa44-CRO (91,411:MH430883) were performed by BLAST analysis. Blue, the gene involved in the replication process; red, antimicrobial resistance-associated genes; yellow, genes associated with transposases and integrases. The degree of genetic simllarity between the six plasmids is depicted by the shade area, and a scale indicating the degree of similarity is at the bottom right.


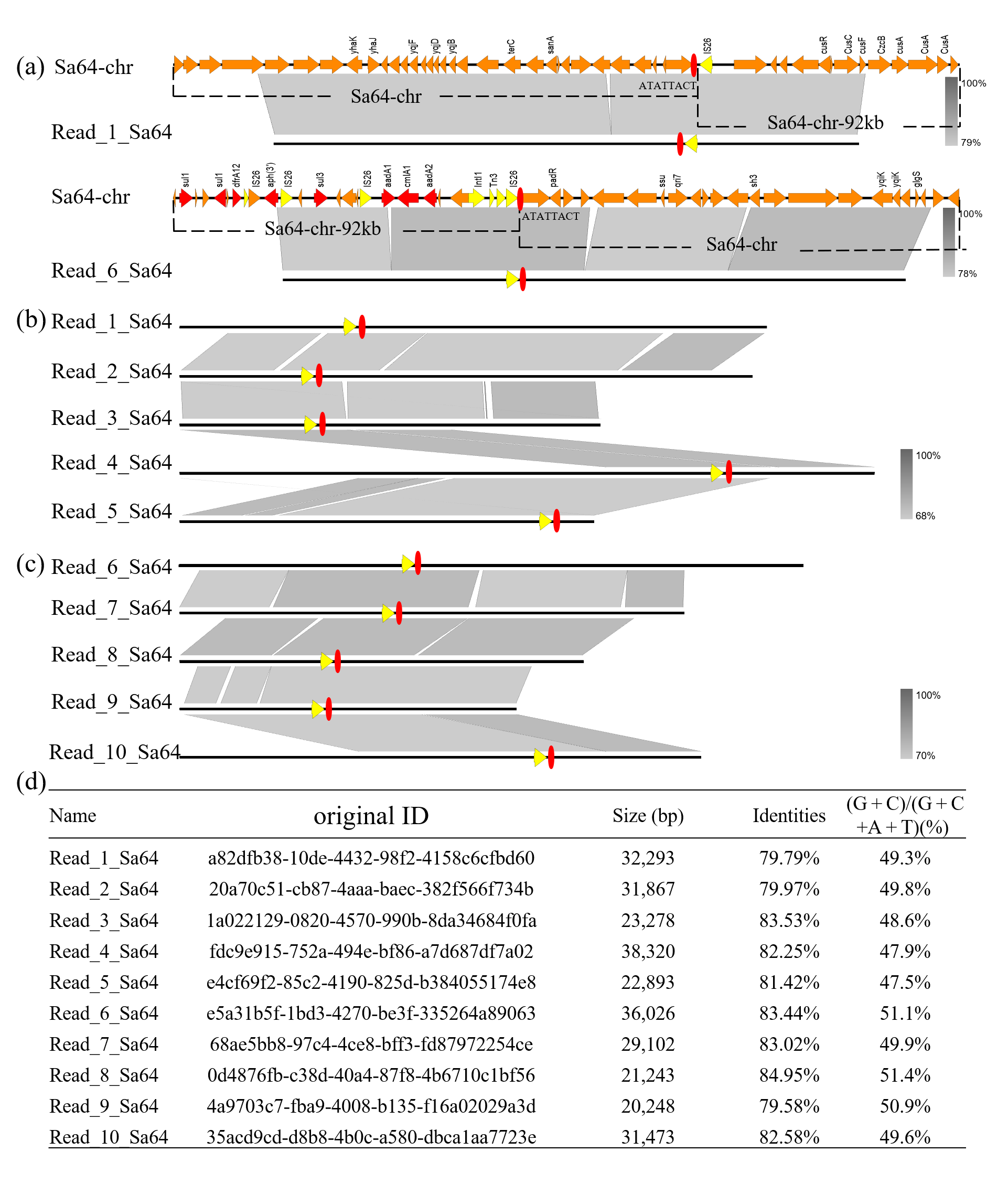
**Supplementary Figure S3.** Nanopore reads covering the fusion region of Sa64-chr and Sa64-chr-92kb. (a) Linear alignment between part of Sa64-chr and the Nanopore long-read Read_1_Sa64 and Read_6_Sa64 at two fusion regions. (b,c)Linear alignment among different Nanopore reads that covering fusion regions of Sa64-chr and Sa64-chr-92kb,yellow arrow depicts IS26 and red denotes hot spot (ATATTACT). (d) Detail information of 10 Nanopore long reads.
